# How Parents Cope with the Care of a Child with Epilepsy: Based upon Grounded Theory

**DOI:** 10.4314/ejhs.v31i2.16

**Published:** 2021-03

**Authors:** Behnaz Bagherian, Monirsadat Nematollahi, Roghayeh Mehdipour-Rabori

**Affiliations:** 1 Assistant Professor, Nursing Research Center, Kerman University of Medical Sciences, Kerman, Iran

**Keywords:** Epilepsy, grounded theory, adaptation process, children, parents

## Abstract

**Background:**

Parents of children with seizure face the complicated health issues of their children. Adaptation strategies of parents as major care providers impact not only their handling of stresses on themselves but also children's quality of life. This study investigated the adaptation processes of parents of children with seizure at two educational hospitals affiliated with Kerman University of Medical Sciences in Iran.

**Methods:**

Twenty parents (15 mothers and 5 fathers), and three nurses were selected using purposive sampling method. Data was collected using qualitative semi-structured interviews and analyzed using the method suggested by Corbin and Strauss version 2008. The interviews were conducted until thematic saturation was achieved.

**Results:**

The adaptation process had five phases: “Disbelief, Patience on what happened, change to preserve, acceptance of the current situation, and self- empowerment.” In summary, the parents of children with seizure had a 5-phase adaptation strategy. The core category achieved was “continued efforts of parents to restore calm.”

**Conclusion:**

Properly assessing the stresses' resources on parents of children with seizure is necessary. Informing their adaptation strategies may help medical staff and social services to provide more targeted support and promote the balance of the family function.

## Introduction

Epilepsy is the most commonly diagnosed chronic disorder of the nervous system in childhood (1). It is a threatening condition and a medical emergency that results in a high percentage of morbidity and death (2). The point prevalence of active epilepsy is 6.38 per 1000 persons, while the lifetime prevalence is 7.60 per 1000 persons. Due to recent advances in medical science and technology, the prevalence of chronic and impaired seizures in the pediatric population is increasing (3). In Iran, 4.2 out of every 1,000 school children have epilepsy, and 65% of the patients with epilepsy are children and teenagers (4).

The unpredictable and chronic nature of epilepsy can affect the physical and social health of the patients and his or her family (5). Besides, the quality of life of people with epilepsy is lower than that of the general population, and it is less or worse in comparison with the quality of life of other patients, such as those with asthma and diabetes (6). It ultimately leads to problems such as lack of self-esteem, depression, anxiety, social isolation, and fear of death at the time of the attack (7). Reily 2014 reported that the suicide risk in these patients is two times that of the healthy population (8). Also, in the study of in the study of Thomas S et al. autism spectrum disorder (9), cognitive disorders (10) are observed in patients with epilepsy. Although many patients with epilepsy have normal IQs, in general, they are weaker in cognitive functions (8). Jones C 2016 found that epilepsy is considered to be a crisis for the whole family and should be supported to adapt to epileptic attacks. The parents of a sick child feel anxious and guilty, and it ultimately affects the whole family's performance (11).

Also, sickness of the child causes negative attitudes toward themselves, behavioral disorders, confusion, fear about the attacks in some parents. Even they were thinking about the uncertain future of the child, fear of psychological and emotional problems in the child. They have financial difficulties caused by the cost of treatment, fear of having another child with epilepsy, fear of complications of medications, and injuries during the seizure attacks. All these issues and problems are influential in the psychological status of the patients' parents (12) (13).

Parents who take care of the epileptic child are emotionally injured, and their routine life is changed. Caregivers of epileptic children may hesitate to spend time away from home because of the fear that the child may have an attack at any moment, and the home environment can provide a secure structure. Therefore, families are more likely to spend time at home (14). Because of the new situation, the parents have to adapt. Also, adaptation is a process in which an individual attempts to recreate a balance in his life through a series of stress-coping behaviors in response to a stressful incidence (15). The nurses can help families to adapt appropriately. These difficulties can be reduced through the support given to these families (16). In Iran, sources of support are limited, and patients suffering from chronic diseases are entirely dependent on their families (16). Furthermore, Iranian patients receive home nursing care that is not enough.

Providing psychological, financial, informational support by health providers help these families adapt faster (17). Understanding the cultural experiences and desires of parents, therefore, is essential to enable one to offer appropriate nursing care. In many cases, hints can be found in the personal experiences of patients and their families, the knowledge of which can help nurses offer services tailored toward preventing the patient's concerns by accurately identifying his or her needs and worries.

Previous studies have investigated only one dimension of the life of the parents of children with epilepsy, and according to the review of the literature, no study was found regarding the adaptation process of parents of children with seizure. Also, parents' adaptation process depends on their attitude, level of education, their own culture, and the number of resources available. A deep understanding of parents' adaptation process helped the health professionals to identify the needs of these children and their families. In this article, we use a qualitative grounded theory approach to explore the process of adaptation process in parents of children with epilepsy.

## Methods

The current study was done using a qualitative approach and grounded theory for identifying the adaptation process of the parents of children with epilepsy. Grounded theory methodology has been an integral part of health social science. It allows for the systematic collection and analysis of qualitative data to inductively develop middlerange theories to make sense of people's actions and experiences in the social world (18).

Qualitative grounded theory is an appropriate method to obtain variable and reliable results from textual data. This method is used to create new knowledge and ideas, and provide context-based facts and guidelines, aiming to condense a broadly described phenomenon, with achievements of deep understanding of the phenomenon (19).

This study was conducted in Kerman, Iran, from June 2016 to October 2019. The city of Kerman is located in the southeastern part of Iran in an area with cultural diversity and mixed ethnicity. The extent of Kerman province, the dispersion of its population, and the high prevalence of epilepsy in this province in conjunction with deficiencies in healthcare services caused the parents of these children to face many problems. In this study, participants included parents of children who have epilepsy coming to the specialized neurological wards of two educational hospitals affiliated with Kerman University of Medical Sciences.

**Participants**: Purposive sampling was used to select participants for this study. The population comprised 15 mothers and five fathers of children with epilepsy admitted to hospitals affiliated with Kerman University of Medical Sciences, Iran, and three nurses working in the neurology and pediatric wards.

The researchers attempted to observe maximum variations in terms of demographic characteristics (numbers of child, level education, and economic situation) and type of epilepsy. The researchers also tried to interview knowledgeable parents who could deliver broad insight into their responses to the study question. The first interviewee was selected on the basis of her experiences to care her child with seizure; she was knowledgeable. The inclusion criteria included the ability to speak the Persian language, the child's age being between one year and 14 years, diagnosis of child's disease at least one year previous to the study, and the participants' willingness to participate in this study. The exclusion criteria were cognitive impairment confirmed by a psychiatrist.

**Data collection**: Data was collected through face-to-face interviews conducted by a Ph.D. holder in nursing that was a research team member. Team members were three assistant professors and a BSc student of nursing. Interview locations and times were agreed upon by both the interviewer and the interviewee. Written and verbal consent was obtained from each participant by the researcher.

First, the researcher told the participants about the study's aims and explained its benefits. Then, based on the study's objectives, general questions were raised. Several examples are as follows: How do you manage your sick child? Please, explain one day of care for your child? How do you solve your problems? (Table 1).

**Table 1:** Interview guide

Questions
What was your child's disease effect on your life?
Please Explain about your experiences of life with your sick child.
How do you manage your child's disease?
How to deal with your child's problems?

The interviews were conducted either at the hospital or the home of the participant and lasted between 30 and 90 minutes, with an average duration of 60 minutes.

**Data analysis:** In the present study, grounded theory method, Corbin and Strauss version 2008, was used for analysis. First, the first author listened to the recorded interviews 4–5 times. In the next step, the whole conversation was typed word-by-word in a Microsoft word document and analyzed using MAXQDA software 10. The interview was transcribed and regarded as a unit of analysis. For better understanding, every finalized document was read by one of the members of the research team, and the meaning units were extracted. The meaning units were categorized and summarized based on similarities and differences, and the meaning codes were extracted. According to the degree of relatedness among meaning codes, they were classified into subcategories that represented the same subject. The assessment of interrelations among subcategories was done, and the main concepts were extracted from them. At the end of each step, the core category was extracted and discussed among research group members. The final findings were discussed with the participants in a meeting, and their last remarks were received.

**Rigor**: For assuring the credibility of the data, we tried to establish a close relationship and a positive interaction with the participants and encourage them to an extensive collaboration. Moreover, we used the ideas and reviews of colleagues, experts, and constant comparisons. We tried to provide the dependability of the findings through constant revisions by experts and participants as well as external observers. For promoting confirmability of the data, we made a great effort to avoid any personal judgment and experiences. For having the maximum transferability of the results, we tried to explain the data as much as possible.

**Ethical considerations**: Ethical considerations were addressed before the study began. All participants completed written informed consent forms and were assured that their information would remain confidential. This study was approved by the Ethics Committee of Kerman University of Medical Sciences (IR.KMU.REC97000448). Study purposes, the confidentiality of data, and recording of interviews were clarified for the participants before their interviews, and their verbal agreements were obtained.

## Results

Of the 23 study participants, fifteen mothers, five fathers of children with epilepsy, and three nurses were interviewed. The interviews were conducted twice for three mothers (26 interviews in total). The range of age for participants was 28–52 years (average 33.5±2.2 years). The interviewed parents all had children with epilepsy of different intensity (generalized epilepsy, focal epilepsy, and generalized and focal epilepsy), and various treatment (types of drugs) were being administered in hospitals. Any patients did not have drug- resistance epilepsy, and all the children studied had only epilepsy, not any other disease. After analyzing the data, the adaptation process of parents in living with children suffering from epilepsy was described.

In this study, parents of children tried to prepare an excellent situation for their child. They were using all available resources. Although the lack of resources and support, as well as their child's care, were excessive burdens on them, they resolved these problems by searching for different strategies and using them to manage care for their children in a peaceful state and to get the best results. The adaptation process had five phases: disbelief, patience on what happened**,** change to preserve, acceptance of the current situation, and self-empowerment.

In summary, parents of children with seizure have a 5-phase adaptation strategy. The core category is achieved, “continued efforts of parents to restore calm” (Table 2).

**Table 2:** Core category, main categories, and subcategories of study

Coping process of parents of children with seizure		

Core category	Main category	subcategory
**continued efforts of** **parents to restore calm**	Disbelief	Denial of the child's illness Escape from reality looking for guilt
	Patience on what happened	Caring patiently
	Change to preserve	Imposing the pressure of the situation on oneself changing the life routine.
	Acceptance of current situation	acceptance of the child's situation by the parents'
		acceptance of the parents' limitations by the child
	Self-empowerment	Self-empowerment of the child
		Self-empowerment of the parents

1. **Disbelief**: The parents of these children did not believe their child's disease. This category consisted of three subcategories “denial of the child's illness, escape from reality, looking for guilt.”

**1.1. Denial of the child's illness:** Almost the parents of these children, in the first phase of the diagnosis of disease, did not accept their situation.

Participant no.23 said, *“After the diagnosis, I did not think that. I took my child to this doctor and that doctor. Maybe one of them rejected the diagnosis. My husband told me, ‘This child did not have any problem. He is healthy. Not take him to the doctor’*”.

**1.2. Escape from reality**: Some of the parents did not follow to treat their sick children. They did not do anything about the definite diagnosis and treatment. They often referred to the time that the child's condition was terrible, and the symptoms of the disease were aggravated.

This subject caused more severe psychological complications for the family and made the treatment more difficult and complicated.

Participant no.21 said, “*Some parents did not want to accept their child's disease. They did not want to accept reality. They wanted to achieve new things. A new diagnosis. For this reason, they changed the doctor”.*

**1.3. Looking for guilt**: feeling guilty and guilty of participants, after the definite diagnosis, the parents sought to find the culprit behind their child's problem.

Participant no 5 said, “*I told God, what you do my God, what did you get from this kid? On the night of winter, I swore allegiance to God. I said, [If I'm guilty why my child. You have to punish me”.*

**2. Patience on what happened:** this category included two subcategories: “**caring patiently**” and “**endure of great sufferings** ”. Almost all of the participants in this experiment tried to be patient throughout every stage of their children's illness and treatment; they tried to tolerate the tension inherent in the situation.

**2.1. “Caring patiently”**: The parents patiently nursed their sick children at all stages and endured many hardships during their children's illness.

Participant No. 4 said: “*When I was breastfeeding my child, I did not rush him to suck and drink faster, because he didn't have enough strength to suck and he was breathing slowly. So I needed to be patient”.*

Participant no. 10 said, “*Because of my bad financial situation, it took a while for me to take my child to the hospital, which was tough”.*

**2-2: Endure of great sufferings:** In this study, all the parents mentioned directly or indirectly the problems that they had to face, and the burdens that they carried throughout this period. Regarding this, participant no. 1 said, “*I locked myself up at home for at least six months, because I didn't want my child to be exposed to any disease, and if I attended even a small party, I would make sure that no one had a cold”.*

Participant no. 6 said, “*I had a difficult situation last year because my child's situation wasn't clear. I didn't know what to do with him when I got to work. When I wanted to take him to kindergarten, it was tough for him. It was a hard time. I did not know what to do. I don't want to experience those days again”.*

**3. Change to preserve:** In this study, change to preserve included two sub-themes: first, imposing the pressure of the situation on oneself, and second, changing the life routine.

**3-1. Imposing the pressure of the situation on oneself**: Parents experienced a difficult situation after their child fell sick. They had to endure some problems related to this particular condition. Many cases studied, parents endure some problems, because there were no other solutions to the problem or because they were unable to do anything other than tolerate the situation.

Participant no. 6 said, “*For a few days when I took my child to kindergarten in the morning, her teacher would say that she would not give her medicine. I had to wake her up early in the morning and give her the medicine, then take her to kindergarten. Normally I would have never awakened her from her sleep, even if I had an important job to take care of. I would let her sleep*”.

Participant no. 2 said, “*I always made healthy, nourishing food for my child. I had to make it. It was my responsibility. I forced myself to make fresh food for him every day”.*

**3-2. Changing the life routine:** Because of their child's illness and need for care, the parents tried to adjust their life to give themselves more opportunities to spend time with their child, even if they had to leave her education or her job.

Participant no. 6 said, “*I was so scared that something would happen to my child at kindergarten. Finally, after thinking a lot, I decided to consult with her doctor. Her doctor wrote a letter for my workplace that my child couldn't be left at kindergarten and that due to her special condition and problems, emotional attachment, and psychological dependence, she may get hurt. So I got three years of unpaid leave”.*

Participant no. Five said, *“After my child got sick, I couldn't work”.*

**4. Acceptance of the current situation:** In this study, acceptance of the situation consisted of two sub-themes: acceptance of the child's situation by the parents and acceptance of the parents' limitations by the child.

**4-1. Acceptance of the child's situation by the parents:** In this stage, the parents accepted their child's illness, inabilities, and limitations after some emotional ups and downs.

Participant no. 3 said, *“Even when we go to a wedding, we return home very early or I stay home with the child because she gets so tired later at night. I don't want any pressure or stress on her. She is not a normal kid; she needs special care. When she gets upset or angry, her appearance was changed”.*

**4.2. Acceptance of the parents' limitations by the sick children**: usually acquire the ability to perform some task by the age of 5 – 6 years. When they suffer from shortness of breath or weakness, and at the beginning of a seizure attack, they can manage the symptoms, stop the task they are doing, and go to a safe place. They cooperate well with their mothers and fathers in this matter, especially those mothers who always speak to their children, give them instructions, and educate them regarding their situation. Participant no 2 said: “*When he goes to play with other kids, he takes them home to play Ludo or computer games instead of going out to run with them. He realizes that running makes him feel bad”.*

**5. Self-empowerment:** Self-empowerment in this study included self-empowerment of the children in order to perform self-related caring activities and usage of resources and possibilities by parents for the self-empowerment of themselves.

**5.1. Self-empowerment of the child:** Children usually become aware of their limitations from 5–6 years old, and they could manage their situation. Mental preparation by their mothers is created caused the Children to have more cooperative with parents. They always talked to their child a bout methods of self-protection and provided the necessary training to them.

Participant (13) expressed, “*my child always knows when he should rest when he is running or which activities will make him feel bad. I have ever trained him. I tell him not to do some exercises. I provide information to him. I always say to him lets search together. I still talk to him and remind him about care tips”.*

Participant (14) expressed*, “Now he can search for things related to his disease or problems which have shaped up in his mind because he has gotten older and he always searches for the answers of questions in the field of his illness.”*

**5.2. Self-empowerment of the parents:** Parents in this study can try for the self-empowerment of themselves using resources and facilities which exist for caring of the child. They tried to provide useful and practical care using consultation with professional people (nurses, doctors), the application of inventiveness, obtaining information from intelligence sources (books, internet), and using others' experiences.

Participant (6) expressed “*My husband and I visited Psychologist a few times before because we felt we should have proper behavior toward the child. I asked my questions from Psychologist about what should I do with this child and how should I behave so that he does not become capricious and also will not have stress. I paid attention to the recommendations of the psychologist and carried out all of them”.*

## Discussion

A total of 23 participants (26 interviews) participated in the study. Fifteen were mothers of children with epilepsy, five were fathers, and three were nurses of the pediatric ward. Interviewed parents had children with epilepsy disease in different types, and they were under treatment with various medicines. Disbelief, patience on what happened, change to preserve, acceptance of the current situation, self-empowerment appeared by analyzing data.

In this section, the available scientific evidence on the importance of codes will be discussed. Although a few qualitative articles were found, we try to use both qualitative and quantitative studies in the discussion.

In this study, at the first diagnosis, some parents did not accept their children's disease and escape from reality. Helges showed that facing a child's illness is a crisis for parents, especially for the mother, because she expects to have a healthy child but suddenly encounters a lot of stress after hearing about the child's illness (20). Also, Burns and Pop mentioned that the parents' first reaction is to deny the child's disease. Besides, feeling guilty and looking for something to blame is another reaction of parents early on after diagnosis (21,22). Although these reactions are normal, parents at this stage need psychological support to be able to go through this phase. Nurses, as crucial members of the care team, should be able to communicate appropriately with parents at a critical stage (23).

However, in the time of child disease diagnosis, parents face a lack of resources in Iran; therefore, families manage to take care of their children with no spiritual, social, or financial support, which in return reduces the quality of life of those children's mothers and fathers (24). As the quality of life of the parents comes down, optimal care and the quality of nursing also decrease (25).

In this study, parents tried to increase their resistance against the problems during the treatment process, and they had different mental capacities. Also, some parents adapted faster, and others were not adapted over time. For example, after three or four years of illness, some parents were still asking themselves why they and why their child? Many parents said they tried to be patient with their child's situation. In this study, all the participants were Muslims who believe in patience. They considered religious teachings, which suggest that human beings must have patience in the face of problems and that what God wills will happen (26). Among the parents in this study, those who had stronger religious beliefs were more calm and relaxed. The majority of the parents increased their mental capacity and patience by going to mosques and attending spiritual programs. Previous studies have also found that praying and going to church are ways of finding peace (27,28). Whaley and Wong pointed out that some families believe that every problem is a way to further grace and faith (26). All the parents who participated in this study had experienced the patient. In a survey of parents' adaptation strategies for Chinese children awaiting a liver transplant, patient care was represented as an experience by their parents (27).

During the adaptation process, parents of sick children made changes to their lives that were necessary to manage life's child. These parents tried to change their routine life to taking good care of their children. Also, they tolerate some problems such as difficulties with giving medicine to their children or their child's fussiness. Thus, nurses should consider these subjects when interacting with these mothers.

Concerning the illness treatments with financial difficulties, problems regarding managing physical symptoms beside performing the duties of their jobs, a lack of knowledge about care, and issues in kindergarten and school were listed as the most challenging problems in managing patients with seizure in both this study and similar others (29–33).

Providing conditions for schools and kindergartens to collaborate more (in the presence of nurses with academic studies) will allow the children to spend a few hours with their peers. This is significantly effective in giving mothers peace of mind and in boosting the well-being and happiness of the children.

Level of stress, anxiety, and depression are higher among children with chronic diseases compared with healthy children (34). Therefore, it is necessary to consider their psychosocial health as well as their physical health (35). Psychosocial care planning for this group of children will be available with a secure support system and practical, accompanied by the family of the child. Educating the children and their families on how to communicate with their peers can reduce the number of problems for them (34). In this study, some mothers had the issue of coordinating work hours with caring for their sick children. Also, some of them had to leave work to take care of their children. However, if conditions in which they could work part-time or perform their job duties at home could be provided, they will be helped to tolerate the situation better, and their mental strength will be increased. The improved maternal mood can help these mothers adjust faster to the harsh conditions of childcare. According to some studies, a job for the mother leads to greater independence, a sense of self-empowerment, enhanced care and commitment, and a longer life expectancy (35).

After the change to preserve, some parents accepted their situation. They tried to provide useful and practical care using consultation with professional people (nurses, doctors), obtaining information from intelligence sources (books, internet), and using others' experiences. Also, they tried to interact with each other, and they learned to care for one another.

Some cases used specific innovations to improve their child's condition, such as connecting with their child's peers to ask them to take care of their children at school or purchasing brain games that do not require physical activity.

In most cases, mothers could deal with the situations created by the disease. For instance, they said that they are in a better mental state in the surgical stage or follow-up stage, after going through the crisis and facing severe conditions (36).

Having a knowledgeable nursing staff empowers mothers (21), and help them to manage the situation. It is one essential need for mothers in Iran. The practical nursing interventions (22) and the availability of nurses at all hours, especially at times when the child feels sick, is essential to adapt faster. Several studies have shown that telenursing systems are significantly useful in creating peace and improving the mental state of chronic patients and their families. Telenursing systems reduce financial costs, eliminate problems regarding long-distance trips, reduce the number of patients referring, and improve patients' quality of life (37). However in Iran, there are no telemedicine systems.

Encouraging parents to take care of themselves is essential, because they will be able to provide more quality care to their children, and the nurses should consider this. The results of some experiments suggest that the health of parents has a significant effect on the physical and psychological recovery of their sick child (38).

In this study, the parents of these children try to adapt themselves to their situations by doing particular behaviors. A deep understanding of the adaptation process can help nurses to complete the identification of the needs of these children and their parents and enables nurses to be more aware of the proper management of the disease. Therefore, parents feel more support, gain more knowledge and information. They achieve faster adaptation and, consequently, more accurate care. Also, a deep understanding of the methods of compatibility can be used to promote the lives of other children and help parents to provide proper care.

In the current study, after passing different stages of stress and anxiety during diagnosis and treatment, the families and the sick children concluded that they should accept and get used to the disease, disabilities, limitations, and repeated visits to doctors.

Despite the mechanisms applied to enhance the rigor of this study, some restrictions may be inherent. The sample size was small, and the context was confined to a particular geographic location. However, the study offers some valuable insights into the ways that parents cope with their situations. The findings of this study can be applied to families and pediatric nurses in other contexts.

The current study is a grounded study. The methodology enabled the investigators to obtain detailed information from the subjects and offer phenomenal explanations. This approach can also unravel answers and lead to a better understanding of existing issues. This study found that parents play a critical role in the care of children with seizure, and they face multiple challenges. It was found that the mothers and fathers in this study went through five phases of coping with their situations and applied adaptation strategies to minimize stress or reduce familial conflicts. Furthermore, determining parents' adaptation strategies will help provide families with targeted interventions to improve family function. Also, the nurses and physicians understand the path of the adaptation process. Additionally, they achieved information about the needs of these children and their parents by this study and helped them to solve their problems.
